# Genomic risk prediction for depression in a large prospective study of older adults of European descent

**DOI:** 10.1038/s41380-025-03145-3

**Published:** 2025-08-06

**Authors:** Chenglong Yu, Andrew Bakshi, Bruno Agustini, Alicia Walker, Tian Lin, Mojtaba Lotfaliany, Lana J. Williams, John J. McNeil, Naomi R. Wray, Michael Berk, Paul Lacaze

**Affiliations:** 1 School of Public Health and Preventive Medicine, Monash University, Melbourne, Victoria Australia; 2 Deakin University School of Medicine, The Institute for Mental and Physical Health and Clinical Translation (IMPACT), Geelong, Victoria Australia; 3 Institute for Molecular Bioscience, The University of Queensland, Brisbane, Queensland Australia; 4 Department of Psychiatry, University of Oxford, Oxford, UK

**Keywords:** Predictive markers, Depression

## Abstract

The extent to which genetic predisposition contributes to late-life depression risk, particularly after age 70, remains unclear, despite the high prevalence of depression in this age group and the variability in risk factors by age. This study investigated the association between a polygenic score (PGS) and depression outcomes, including severity, trajectories of depression, and antidepressant medication use, in a longitudinal cohort of 12,029 genotyped older adults of European descent aged ≥70 years, with no history of diagnosed cardiovascular disease events, dementia, or permanent physical disability at baseline. Participants were followed for a median of 4.7 years. The PGS was derived using the latest Psychiatric Genomics Consortium data for major depression. Depression was defined by the CES-D-10 score thresholds of ≥8 (primary outcome), ≥10, and ≥12 (secondary outcomes), alongside antidepressant medication use and four previously established longitudinal trajectories of depressive symptoms: low (non-depressed), moderate (subthreshold), high (persistent), and initially low but increasing (emerging). Multivariable models were used to examine associations between the PGS (per standard deviation, SD) and outcomes, adjusting for covariates. At baseline, mean participant age was 75.1 years, 54.9% were female, and 9.1% had depression (CES-D-10 ≥ 8). The PGS was significantly associated with baseline depression (OR = 1.23 [1.15–1.31]), incident depression (HR = 1.18 [1.14–1.23]) and antidepressant medication use (OR = 1.39 [1.31–1.47]). Compared with non-depressed participants, the PGS was associated with increasing severity of depression trajectory classes (subthreshold depression OR = 1.15 [1.11–1.20], emerging depression OR = 1.22 [1.13–1.31], persistent depression OR = 1.40 [1.31–1.49]). These findings suggest that the PGS may play an important role in risk stratification for late-life depression.

## Introduction

Depression is a complex disease influenced by both genetic and environmental factors [1, 2]. Genetic predisposition plays a meaningful role in depression, as indicated by twin and family studies, with heritability estimates ranging from 30–40% [3]. However, as the mix of risk factors varies by age, the extent to which genetic predisposition contributes to late-life depression (after age 70 years) is less known, despite the prevalence of depression being particularly high in this age group [4]. Understanding the role that genetics may play in late-life depression could help enable early recognition and early intervention, potentially improving prognosis, risk stratification and management of depression for this population [5–9].

Recent genome-wide association studies (GWAS) have identified an increasing number of single nucleotide polymorphisms (SNPs) associated with major depression [10–15]. These depression-associated common variants can be used to calculate a polygenic score (PGS), acting as a single measure of aggregate genetic risk from many common variants [16, 17]. PGSs have been shown to be associated with clinically relevant depression [18–20]. However, few studies have evaluated the performance of a PGS in late-life depression specifically for individuals aged 70 and older [21, 22]. No studies, to our knowledge, have investigated how a PGS correlates with the longitudinal course, trajectories and prognosis of depression in this older age group.

Depression in older people is very common both in population and primary care samples [23]. It is particularly common in people with chronic medical comorbidity and women, where recent stressful life events, social isolation, functional decline, emerging medical comorbidity and symptoms like insomnia can serve as risk factors [24]. It is frequently undetected or undertreated in primary care, particularly in minority groups and males. Comorbidity with chronic medical disorders, pain, polypharmacy, early-onset cognitive decline and substance abuse complicate the detection and management of depression in older people [25–27]. If untreated, late-life depression is associated with poorer social and physical functioning and decreased quality of life, poorer adherence to treatment, and the aggravation of chronic medical comorbidities, contributing to increased morbidity and mortality in this population [28].

The largest-ever GWAS of major depression was recently undertaken by the Psychiatric Genomics Consortium (PGC), comprising 685,808 cases and 4,364,225 controls from 109 cohorts [15]. This PGC GWAS identified 697 independent associations at 636 loci (293 of them are novel) with depression at genome-wide significance (P < 5 × 10^−8^) [15]. The newly-associated variants can now be incorporated into an updated and improved PGS for depression. The goal of our study was to independently assess and validate the performance of this new PGS in a longitudinal cohort of older adults aged ≥70 years, to specifically investigate performance for late-life depression. Further, our study sought to examine the extent to which genetic susceptibility for depression (measured using the PGS) was associated with different depression trajectories, in a well-characterised prospective study of older adults enrolled into the ASPirin in Reducing Events in the Elderly (ASPREE) randomized controlled trial [29–31].

## Methods

### Study population

ASPREE is a randomized double-blind placebo-controlled clinical trial, which aimed to determine whether daily 100-mg aspirin extended disability-free survival in healthy adults aged 70 years and older (65 years of age and older for U.S. participants) with no history of diagnosed cardiovascular diseases, dementia, physical disability, or other life-threatening illness at enrolment [32]. The design and protocol of the ASPREE trial have been reported previously [29–31]. All participants provided written informed consent. The study was registered on ClinicalTrials.gov (identifier: NCT01038583) and approved by the Alfred Hospital Human Research Ethics Committee in Australia and site-specific Institutional Review Boards in the United States. All methods were performed in accordance with the relevant guidelines and regulations.

In this study, we included only genotyped participants of European descent from the ASPREE trial (Fig. S1). We excluded participants without imputed genotype data available, close relatives with a genetic relationship >0.05 using the GCTA package [33] and participants without a baseline CES-D-10 (Center for Epidemiologic Studies Depression Scale 10) score. We also excluded participants with non-European ancestry to reduce the effect of population stratification bias on the PGS distribution. These individuals were identified through genetic principal component (PC) analysis and matched with the European subset of the 1000 Genomes Project phase 3 reference population (see Fig. S2 for a PC plot of ethnicities). Furthermore, the non-European ancestry samples (N = 534, representing 4% of all genotyped participants) lacked sufficient power to evaluate PGS performance or conduct subgroup analyses in an ancestry-specific manner. Out of the 19,114 ASPREE trial participants, a total of 12,029 genotyped, unrelated, European-ancestry participants were included in the study.

### Outcomes

ASPREE participants were evaluated for depressive symptoms (in the past week) at baseline and up to six annual follow-up visits during their participation in the ASPREE trial using the CES-D-10 (Table S1). The CES-D-10 is a validated, self-rated questionnaire with a single-factor structure ranging from 0 to 30, with higher scores indicating a greater depression level (Fig. S3) [4, 34]. Depression was defined as a CES-D-10 score of ≥8 and more stringent thresholds of ≥10 and ≥12 were also applied based on previous studies [35].

For baseline depression, we divided participants into cases and controls using the three CES-D-10 score thresholds ( ≥ 8, ≥10, and ≥12). For incident depression, we focused on the controls at baseline (without depression) and examined their annual follow-up CES-D-10 scores. Incident depression was defined as reaching the CES-D-10 score of ≥8 for the first time at an annual follow-up [4].

We then examined the association between the PGS and four distinct depression trajectory classes, previously identified using latent class mixed models (LCMMs) in the ASPREE trial [36]. Examination of the symptoms associated with these trajectories suggest that they represent different longitudinal patterns of depressive symptoms: non-depressed (n = 5536; consistently low symptoms), subthreshold depression (n = 4625; consistently moderate symptoms), persistent depression (n = 1089; consistently high symptoms), and emerging depression (n = 779; initially low but increasing symptoms over time). Depressive symptoms were modelled using repeated CES-D-10 scores collected at baseline and annual follow-ups. LCMMs captured curvilinear patterns of symptom progression, classifying participants into distinct groups based on their longitudinal symptom trajectories. The final model selection was determined by statistical fit indices, including the Akaike information criterion, Bayesian information criterion, entropy, and log-likelihood values, ensuring optimal classification of participants into meaningful trajectory groups [36].

Antidepressant medication (ATC code of “N06A”) use at baseline was also examined as an outcome.

### Genotyping and calculation of the PGS

DNA samples provided by the ASPREE Biobank were genotyped using the Axiom 2.0 Precision Medicine Diversity Research Array (Thermo Fisher Scientific, CA) as described previously [37, 38]. Variant calling used a custom pipeline aligned to the human reference genome GRCh38, and imputation was performed using the TOPMed Imputation Server with TOPMed-r2 reference panel [39, 40]. Quality control removed variants with low imputation quality scores (r^2^ < 0.3).

PGS were generated for the 12,029 ASPREE participants using ~7 million SNPs with weights derived from the SBayesRC method [41] applied to the latest major depression GWAS summary statistics from the PGC [15]. SBayesRC is a new PGS method which extends the previous SBayesR [42] to incorporate functional annotations and allows for the joint analysis of all common SNPs in the genome [41]. We used the GCTB software-recommended linkage disequilibrium reference sample to infer the expected correlation structures between SNP associations statistics (https://cnsgenomics.com/software/gctb). In the PGC GWAS paper [15], the authors also reported a SBayesR leave-one-cohort-out PGS using 42 European-ancestry cohorts with individual level data, providing a useful benchmark to the results provided here.

The full GWAS summary statistics for the 23andMe discovery data set will be made available through 23andMe to qualified researchers under an agreement with 23andMe that protects the privacy of the 23andMe participants. Datasets will be made available at no cost for academic use. Please visit https://research.23andme.com/collaborate/#dataset-access/ for more information and to apply to access the data.

### Statistical analyses

We assessed associations between PGS and baseline depression (CES-D-10 score of ≥8), using logistic regression models to estimate the odds ratio (OR) and 95% confidence intervals (CI), expressed per standard deviation (SD) of the PGS. The first model (Model 1.1) was adjusted for age at enrolment, sex, and the first 20 ancestry PCs to account for population structures, and a second model (Model 1.2) was additionally adjusted for other covariates including living status, educational attainment, smoking status, alcohol drinking, and body mass index. These covariates were described previously [30]. To evaluate the association of the PGS with baseline depression, we also estimated the area under the receiver operating characteristic curve (AUC) for a model containing non-genetic risk factors (Model 1.2). The improvement in AUC after adding the PGS to the model was assessed using the DeLong test to compare nested models.

Association between the PGS and antidepressant use at baseline was assessed using Models 1.1 and 1.2. We applied Cox proportional hazards models (Models 2.1 and 2.2), using the same covariate adjustments as in models 1.1 and 1.2, to estimate hazard ratios (HR) and 95% CI per SD for the associations between PGS and risk of incident depression at annual follow-up visits, using years since randomization as the time-to-event. The PGS included in the models above was also investigated by categorical groups (deciles).

We used multinomial logistic regression (Models 3.1 and 3.2), which were implemented using an R package ‘nnet’ [43], to examine the association (OR and 95% CI per SD) between the continuous PGS and four depression trajectory classes by setting each of the classes as the reference level. In models 3.1 and 3.2, we used the same covariate adjustments as in models 1.1 and 1.2.

We examined sex-specific PGS-associated changes in depression outcomes by including an interaction term PGS-by-sex in the full models above. To evaluate whether lifestyle and educational attainment may modify the genetic effect on depression, we also investigated interactions between PGS and five covariates (living status, smoking status, alcohol drinking, body mass index, and educational attainment). These gene-by-covariate interactions were considered to be significant after Bonferroni correction for multiple comparisons across 13 outcomes and six covariates (corrected P-value = 0.05/(13 × 6) = 0.0006).

## Results

### Baseline characteristics

The baseline characteristics of the study population are shown in Table 1. The mean (SD) age of the participants was 75.1 (4.2), and 6,604 (55%) of the participants were women. Most of the participants lived at home, either alone (32%) or with family, friends or a spouse (68%). Only a small number of participants were current smokers (3%), and most consumed alcohol at baseline (80%).

**Table 1:** Baseline characteristics of participants, stratified by polygenic risk group.

Baseline characteristics	Overall (n = 12,029)	Low polygenic risk (Q1) (n = 2406)	Medium polygenic risk (Q2-4) (n = 7218)	High polygenic risk (Q5) (n = 2405)
Age, mean (SD), years	75.1 (4.2)	75.3 (4.3)	75.1 (4.3)	74.8 (4.0)
Sex, n (%)				
Male	5425 (45%)	1040 (43%)	3310 (46%)	1075 (45%)
Female	6604 (55%)	1366 (57%)	3908 (54%)	1330 (55%)
Educational attainment, n (%)				
< 9 years of education	1840 (15%)	333 (14%)	1134 (16%)	373 (16%)
9–11 years of education	3779 (31%)	749 (31%)	2245 (31%)	785 (33%)
12 years of education	1343 (11%)	274 (11%)	798 (11%)	271 (11%)
13–15 years of education	1898 (16%)	418 (17%)	1133 (16%)	347 (14%)
16 years of education	1105 (9%)	233 (10%)	665 (9%)	207 (9%)
17–21 years of education	2064 (17%)	399 (17%)	1243 (17%)	422 (18%)
Living status, n (%)				
At home alone	3812 (32%)	753 (31%)	2264 (31%)	795 (33%)
At home with family, friends, or a spouse	8171 (68%)	1644 (68%)	4924 (68%)	1603 (67%)
In a residential home^a^	46 (0.4%)	9 (0.4%)	30 (0.4%)	7 (0.3%)
BMI^b^, mean (SD), kg/m^2^	28.0 (4.5)	27.6 (4.3)		28.4 (4.8)
Smoking status, n (%)				
Current	367 (3%)	59 (2%)	217 (3%)	91 (4%)
Former	4989 (41%)	909 (38%)	2997 (42%)	1083 (45%)
Never	6673 (55%)	1438 (60%)	4004 (55%)	1231 (51%)
Alcohol drinking status, n (%)				
Current	9606 (80%)	1985 (83%)	5743 (80%)	1878 (78%)
Former	584 (5%)	76 (3%)	364 (5%)	144 (6%)
Never	1839 (15%)	345 (14%)	1111 (15%)	383 (16%)

^a^Supervised care or assisted living.

^b^BMI, body mass index, calculated as weight in kilograms divided by height in meters squared.

### PGS and baseline characteristics

To evaluate the replication of depression-associated SNPs, we analyzed the effect sizes of the top 1000 independent significant SNPs from PGC (European descent) [15] in the ASPREE cohort. Regression analysis revealed a significant positive correlation (r = 0.20, P = 1.04E-10 for CES-D-10 score of ≥8, r = 0.11, P = 5.35E-04 for CES-D-10 score of ≥10, and r = 0.12, P = 8.93E-05 for CES-D-10 score of ≥12) between effect sizes in the two datasets across different CES-D-10 thresholds (Fig. S4). These findings reinforce the polygenic nature of depression and indicate that the genetic effects identified in PGC are also relevant in an older population.

The PGS was generated using a total of 7,329,199 variants with weights derived from the SBayesRC. Fig. S5 shows the distribution of the PGS for the 12,029 participants. No major differences were observed in baseline characteristics between the PGS risk groups (e.g., low-risk 0–20%, medium-risk 21–80% and high-risk 81–100%) (Table 1).

### PGS and baseline depression

At baseline, there were 1,096 (9.1%) participants with a CES-D-10 score of ≥8, 535 (4.4%) with a CES-D-10 score of ≥10, and 291 (2.4%) with a CES-D-10 score of ≥12. Consistent with the PGC study [15], which adjusted for the top 20 ancestry PCs and assumed a lifetime prevalence of 15%, our PGS explained 1.5% of the variance of depression on the logistic liability scale [44] for CES-D-10 ≥ 8, 3.0% for CES-D-10 ≥ 10, and 5.3% for CES-D-10 ≥ 12 in the ASPREE population. The proportion of variance explained for CES-D-10 ≥ 12 is close to the PGC result (5.8% on the logistic liability scale) in the leave-one-out analyses using 42 European ancestry cohorts [15].

Significant positive associations were observed between the continuous PGS (per SD) and depression at baseline (Table 2). An increasing effect of the PGS was observed with increasing CES-D-10 thresholds (CES-D-10 ≥ 8 adjusted OR = 1.23 [95% CI: 1.15–1.31]; ≥10, OR = 1.28 [1.17–1.40]; ≥12, OR = 1.35 [1.20–1.52] per SD).

**Table 2:** Association of the PGS (as a continuous variable) with baseline and incident depression per different CES-D-10 thresholds.

CES-D-10 threshold	Baseline depression				Incident depression during follow-up			
	Case No.	Control No.	Model 1.2		Incident case No. during follow-up	Non-depressed No. during follow-up	Model 2.2	
			OR (95% CI)	P			HR (95% CI)	P
≥8	1096	10,933	1.23 (1.15–1.31)	2.29E-10	3015	7918	1.18 (1.14–1.23)	9.90E-20
≥10	535	11,494	1.28 (1.17–1.40)	3.64E-08	2121	9373	1.23 (1.17–1.28)	4.69E-20
≥12	291	11,738	1.35 (1.20–1.52)	9.75E-07	1412	10,326	1.30 (1.23–1.37)	2.18E-22

Logistic Model 1.2 and Cox Model 2.2 were used to estimate the odds ratio (OR) or hazard ratio (HR) of the polygenic score (PGS) per standard deviation, with 95% confidence intervals (CI). These models were adjusted for age, sex, the first 20 principal components of genetic ancestry, living status, educational attainment, smoking status, alcohol drinking status, and body mass index.

**Fig. 1:**
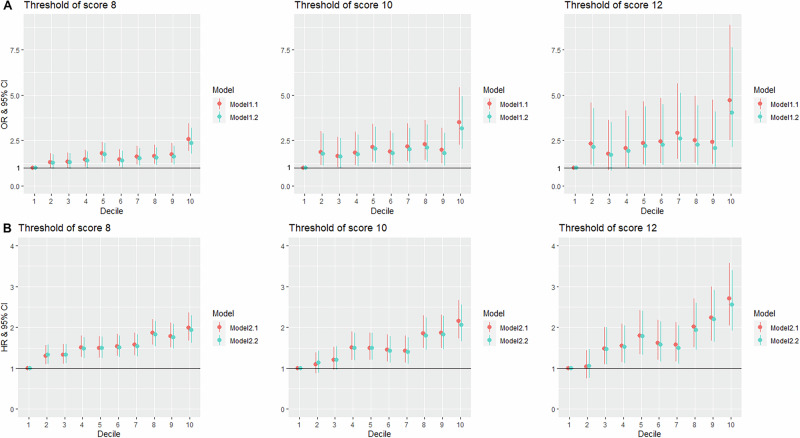
Association of the PGS (as a categorical variable) with baseline and incident depression. Association of PGS decile groups with baseline (**A**) and incident (**B**) depression per CES-D-10 threshold. Logistic models 1.1/1.2 and Cox model 2.1/2.2 were used to estimate the odds ratio (OR) and hazard ratio (HR) of the polygenic score (PGS) as a decile (comparing to the 1^st^ decile), with 95% confidence intervals (CI), respectively. The models 1.1 and 2.1 were adjusted for age, sex, and the first 20 principal components of genetic ancestry. The models 1.2 and 2.2 were additionally adjusted for living status, educational attainment, smoking status, alcohol drinking status, and body mass index.

In Fig. 1A, we present the association between the PGS and depression at baseline, considering the PGS distribution in decile groups. Compared with the 1^st^ PGS decile group as a reference (0–10%, the lowest-risk level), participants in the 10^th^ PGS decile group (90–100%, the highest-risk level) have an approximately 2.4-fold increased risk of depression at baseline, using the CES-D-10 ≥ 8 threshold (adjusted OR = 2.37 [95% CI: 1.77–3.18]). The effect increased when higher CES-D-10 thresholds were applied (≥10, OR = 3.16 [2.03–4.93]; ≥12, OR = 4.04 [2.14–7.62]). These associations became slightly stronger in models (Model 1.1) that did not adjust for educational attainment and lifestyle-related variables (Table S2 and Fig. 1A).

AUC estimates and comparisons for associations between the PGS and baseline depression are presented in Table S3, evaluating the incremental value of PGS beyond non-genetic risk factors. The AUC of the baseline model (including only non-genetic risk factors) for baseline depression at the CES-D-10 ≥ 8 threshold was 0.62 (95% CI: 0.60–0.63). When the PGS was added, the AUC increased to 0.63 (95% CI: 0.61–0.64), with a significant improvement (P = 0.02). A similar pattern was observed with higher CES-D-10 thresholds. For CES-D-10 ≥ 10, the AUC improved from 0.64 to 0.65 (P = 0.02). For CES-D-10 ≥ 12, the AUC increased from 0.67 to 0.69, but this change was not statistically significant (P = 0.09), possibly due to fewer participants endorsing these higher thresholds which limits power despite the higher association/AUC.

### PGS and incident depression

The continuous PGS (per SD) was associated with incident depression during follow-up (Table 2), after removing participants with depression at baseline. The association of the continuous PGS with incident depression was observed with higher effects with increasing specificity of diagnosis (CES-D-10 ≥ 8, adjusted HR = 1.18 [1.14–1.23]; ≥10, HR = 1.23 [1.17–1.28]; ≥12, HR = 1.30 [1.23–1.37] per SD).

In Fig. 1B, we show categorical analysis of the PGS in decile groups with incident depression at annual follow-up visits. Compared with the 1^st^ decile group (0–10%, the lowest-risk level), the 10^th^ decile group (90–100%, the highest-risk level) had approximately two-fold higher risk of incident depression during follow-up (CES-D-10 ≥ 8, adjusted HR = 1.93 [1.62–2.29]; ≥10, HR = 2.06 [1.66–2.55]; ≥12, HR = 2.55 [1.92–3.39]). These associations became slightly stronger in models (Model 2.1) that did not adjust for educational attainment and lifestyle-related variables (Table S2 and Fig. 1B).

### PGS and antidepressant medication use

There were 1345 (11.2%) participants using antidepressant medication at baseline, which was significantly associated with baseline CES-D-10 scores (adjusted OR = 1.14 [1.12–1.15]) and incident depression during follow-up (CES-D-10 ≥ 8, adjusted HR = 2.16 [1.96–2.38]; ≥10, HR = 2.42 [2.17–2.70]; ≥12, HR = 2.78 [2.45–3.15]).

We found a strong association between the PGS (per SD) and antidepressant medication use at baseline (adjusted OR = 1.39 [1.31–1.47], Table S4). The associations between the decile groups and antidepressant medication use are presented in Fig. S6, where the 10^th^ decile group (90–100%) was associated with over three-fold increased risk of anti-depressant medication use, compared with the 1^st^ decile (0–10%) (adjusted OR = 3.60 [2.71–4.80]).

### PGS and trajectory of depressive symptoms

**Fig. 2:**
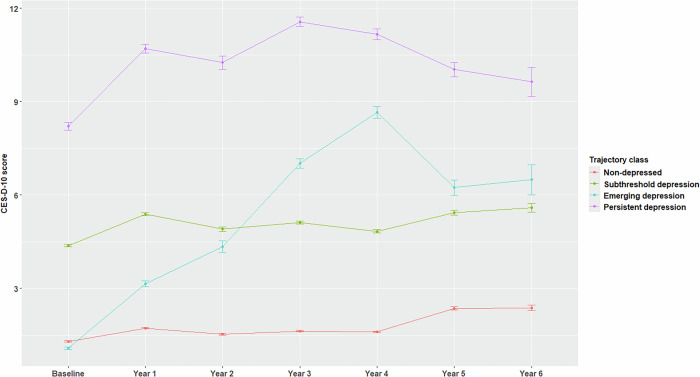
Longitudinal CES-D-10 score (mean and standard error) of four depression trajectory classes. Four distinct classes reflect consistently low (Non-depressed), consistently moderate (Subthreshold depression), consistently high (Persistent depression) and initially low but emerging symptoms of depression (Emerging depression).

In Fig. 2, we show the four distinct longitudinal CES-D-10 trajectory classes, reflecting consistently low, consistently moderate, consistently high and initially low but emerging symptoms of depression, as published previously [36]. We used boxplots (Fig. S7) to show the mean and interquartile range of standardized PGS across the four groups, and we found the increasing means (SD) of −0.10 (0.99), 0.05 (1.00), 0.10 (0.97) and 0.26 (1.01) for non-depressed, subthreshold, emerging and persistent depression groups, respectively.

We examined associations between the continuous PGS (per SD) and the four trajectory classes (Table 3). Using the non-depressed group as a reference, we observed an increasing effect of the PGS with more severe depression trajectory classes: subthreshold depression (adjusted OR = 1.15 [1.11–1.20]); emerging depression (OR = 1.22 [1.13–1.31]); persistent depression (OR = 1.40 [1.31–1.49]). Furthermore, the PGS predicted a higher risk for persistent depression compared with the subthreshold depression class (OR = 1.21 [1.13–1.30] and the emerging depression class (OR = 1.15 [1.05–1.26]). However, the PGS did not distinguish between emerging depression and subthreshold depression with no significant difference between these trajectory classes (OR = 1.06 [0.98–1.14], P = 0.17). These associations became slightly stronger in models (Model 3.1) that did not adjust for educational attainment and lifestyle-related variables (Table S5).

**Table 3:** Association of the PGS (as a continuous variable) with depression trajectory classes.

Depression trajectory class comparison	Model 3.2	
	OR (95% CI)	P
Non-depressed (reference level) vs. Subthreshold depression	1.15 (1.11–1.20)	3.37E-12
Emerging depression	1.22 (1.13–1.31)	5.01E-07
Persistent depression	1.40 (1.31–1.49)	1.62E-22
Subthreshold depression (reference level) vs. Emerging depression	1.06 (0.98–1.14)	0.17
Persistent depression	1.21 (1.13–1.30)	2.45E-08
Emerging depression (reference level) vs. Persistent depression	1.15 (1.05–1.26)	3.91E-03

Non-depressed, n = 5536; Subthreshold depression, n = 4625; Emerging depression, n = 779; Persistent depression, n = 1089. Multinomial logistic Model 3.2 was used to estimate the odds ratio (OR) of the polygenic score (PGS) per standard deviation, with 95% confidence intervals (CI). The model was adjusted for age, sex, the first 20 principal components of genetic ancestry, living status, education attainment, smoking status, alcohol drinking status, and body mass index.

No significant PGS-by-sex interaction was observed (P > 0.05, Table S6), which implies that PGS-associated changes in the depression outcomes were not sex-specific in our data. Several marginally significant signals were detected for PGS-by-covariate interactions on depression outcomes; however, none of them was significant after multiple testing corrections (P > 0.0006, Table S6).

## Discussion

In this study, we evaluated the performance of a recently-derived PGS for major depression in an independent, well-characterised prospective cohort of older adults aged ≥70 years who were relatively healthy at baseline. Our results demonstrate that the PGS is associated with both baseline and incident late-life depression in this older population, with stronger effects of the PGS observed with higher CES-D-10 thresholds. We also found a strong association between the PGS and antidepressant medication use at baseline. Compared with the non-depressed, the PGS had a stepwise increasing effect on subthreshold, emerging and persistent depression trajectories during follow-up - presenting a new way of assessing PGS associations with longitudinal depression phenotypes. The PGS also predicted a higher risk for persistent depression compared with the subthreshold and emerging depression classes. These findings show that genetic predisposition contributes to late-life depression in older people in a dose dependent manner and that associations of the PGS were robust across different thresholds and definitions of depression.

Out-of-sample PGS analyses using 42 cohorts recently reported by the PGC [15] have provided a benchmark to assess the performance of the current PGS and to interpret our results. The proportion of variance explained for depression outcomes (CES-D-10 ≥ 8 or ≥10) by the PGS in our cohort is smaller than that reported in other cohorts by the PGC, which is expected given that CES-D-10 is based on depressive symptoms in the past week. However, using a depression threshold of CES-D-10 ≥ 12, we obtained similar proportions of variance explained (5.3% in ASPREE versus 5.8% in PGC, using the same liability scale [15, 44]). This may imply that a more stringent threshold of CES-D-10 may be closer to a clinical diagnosis of major depressive disorder (MDD). Nonetheless, following the latest recommendations in the field [20], we opted for a lower threshold to increase sensitivity (and thus our sample size) as this score was previously validated in this population [45].

The combined OR for the 10^th^ PGS decile relative to the 1^st^ decile of depression risk reported by the PGC was 4.92 [95% CI: 4.57–5.29] [15]. This effect size is very similar to the maximum effect we observed for the PGS in the ASPREE population of 4.72 [2.51–8.88] using a CES-D-10 ≥ 12 threshold (Model 1.1, Fig. 1A). Noteworthy differences between the ASPREE population and discovery cohorts used in the PGC include: a) ASPREE was a highly selected healthy older population, depleted of unwell people at baseline, b) the definitions of depression used in other genetic studies have varied, and c) the fact that the discovery meta-analysis GWAS performed by the PGC used 42 different cohorts, most of which were younger than ASPREE, and for many of which the participants were either not assessed for or did not meet the criteria for MDD as defined by the DSM-5 [15]. This speaks to the variable definition of depression used in different cohort studies, which will consequently impact the effect size and strength of association of the depression PGS.

In ASPREE, a self-reported CES-D-10 questionnaire was utilized to record depressive symptoms in the past week for the participants prior to each annual visit. The CES-D-10 score, therefore, acts as a proxy measure of MDD, without clinical confirmation or diagnosis, and therefore is not equivalent to other definitions of MDD based on DSM-5 or clinical diagnosis by a psychiatrist. This may contribute to the lower effect size observed in ASPREE, demonstrating that the depth and rigour of phenotyping are important in genetic studies of depression [20, 46]. Moreover, our study included only healthier older adults aged ≥70 years and focused on late-life depression. Late-life versus earlier-life depression may vary based on differential proportions of genetic contributions and the role of age specific environmental risk factors. The older age of the ASPREE cohort is notably different from the age of most cohorts included in the PGC, e.g., the UK Biobank (enrolled age 40 to 69) [47]. The contribution of genetic predisposition to late-life depression may be less pronounced than in other populations, comprised of young [18, 19] or middle-aged [21, 22, 48] people.

Cao et al. [49]. recently used the UK Biobank population to derive a linear and dose-response association between continuous PGS and risk of incident depression (HR = 1.09 [95% CI: 1.06–1.12] per SD). In the ASPREE cohort, we observed stronger genetic effects with CES-D-10 categories (1.18 [1.14–1.23] per SD for ≥8, 1.23 [1.17–1.28] per SD for ≥10, 1.30 [1.23–1.37] per SD for ≥12). Furthermore, our results concur with their conclusion suggesting there is no significant interaction between genetic risk and lifestyle factors, i.e., genetic and lifestyle factors were independently associated with risk of incident depression [49].

We found a significant association between the PGS and antidepressant medication use at baseline in the ASPREE population. Given that use of antidepressants was significantly associated with baseline and incident depression in our data, this finding was expected. The effect of the PGS associated with antidepressant medication use at baseline (OR = 1.39 [95% CI: 1.31–1.47] per SD) was stronger than that observed when associated with CES-D-10 categories (OR = 1.23 [1.15–1.31] per SD for ≥8, 1.28 [1.17–1.40] per SD for ≥10, 1.35 [1.20–1.52) per SD for ≥12], which suggests that genetics may play a significant role in individuals who develop depression earlier in life and are consequently receiving treatment. Furthermore, associations between depression polygenic risk scores and antidepressant treatment response have been reported elsewhere, including in MDD patients [50, 51]. Thus, the PGS could potentially be used to predict antidepressant treatment response, yet this requires further studies with larger sample sizes that include standardized MDD case definitions [50].

Another key feature of our study is the investigation of longitudinal associations between the PGS and depressive symptom trajectories. Longitudinal associations between depression trajectories in later life have been previously associated with poor health-related outcomes such as physical disability, cancer, dementia and major hemorrhage [36]. Our findings shed new light on the relationship between genetic predisposition and late-life depression trajectories and suggest there may be different magnitudes of genetic predisposition contributing to each trajectory class. If true, this may suggest that use of a PGS in the future could help refine risk-stratification strategies for late-life depression, potentially helping to predict the course of depression in selected populations and implement targeted therapeutic strategies.

The strengths of our study include a large sample size of well-characterised, initially-healthy older individuals; a prospective study design with a median follow-up time of 4.7 years; standardized protocols for data collection; and serial measures of CES-D-10 at each annual visit alongside concomitant medication use, which enabled longitudinal analysis of depression at different severity thresholds and distinct trajectories of depressive symptoms.

### Limitations

Firstly, our study was restricted to participants of European ancestry and therefore our findings cannot be generalized to other ancestries. Future studies should validate the PGS in more ancestrally diverse cohorts to ensure its applicability across different populations, and to address potential disparities in genetic prediction models. Secondly, the stringent exclusion criteria for the ASPREE trial at enrolment may have resulted in a healthy survivorship bias, meaning that ASPREE has ascertained a population with lower rates of comorbidity than the general population. This may have resulted in an underestimation of the true effect of the PGS in late-life depression in the general population. Thirdly, while our findings demonstrate robust associations between genetic predisposition and depression in older adults, the biological mechanisms underlying these associations remain unclear and are not well defined. Polygenic scores aggregate the effect of many trait-associated common genetic variants, each of which has a potentially varying biological function or mechanism with regards to depression risk; in fact, the variants may have no causal function at all with the association achieved through correlation with causal variants. Future studies incorporating functional genomics, transcriptomics, and brain imaging data would be needed to understand the functional and neurobiological pathways involved in depression, such as inflammation, neuroplasticity, and synaptic function.

## Conclusions

In summary, a newly-derived PGS for depression is associated with various depression outcomes and phenotypes in a well-characterized longitudinal cohort of older adults. This demonstrates that genetic predisposition to depression persists in older people and contributes to late-life depression. An improved understanding of the associations between genetic versus environmental risk factors for late-life depression may help improve risk stratification approaches. Further clinical studies are required to assess possible implementation, examining potential delivery methods, appropriate settings, and the overall clinical utility of the PGS. Validation in larger, more diverse cohorts is also required to confirm the predictive value of PGS across other populations, particularly in assessing its role in guiding treatment decisions and evaluating potential gene-by-treatment interactions in late-life depression management.

## Supplementary information

Supplemental content

## Data Availability

Data used in this study can be accessed upon request through the ASPREE Principal Investigators. Application details are provided on the ASPREE website (www.ASPREE.org). The data will be made available to investigators whose research proposals have been approved by a designated review committee.

## Code availability

All codes used in the analyses are available upon request to the authors.
