# Supplementary material for: Genomic risk prediction for depression in a large prospective study of older adults of European descent
Source: Mol Psychiatry. 2025 Aug 6;30(12):5865–72. doi: 10.1038/s41380-025-03145-3 (PMC12602314; doi:10.1038/s41380-025-03145-3)
Supplement: Supplementary file 1 — Supplemental content [file 41380_2025_3145_MOESM1_ESM.pdf]

Genomic risk prediction for depression in a large prospective study of older adults of European descent

Supplemental content

This file includes 7 supplementary figures and 6 supplementary tables.

Figure S1: Flow chart of selection of the final dataset in ASPREE for this study.

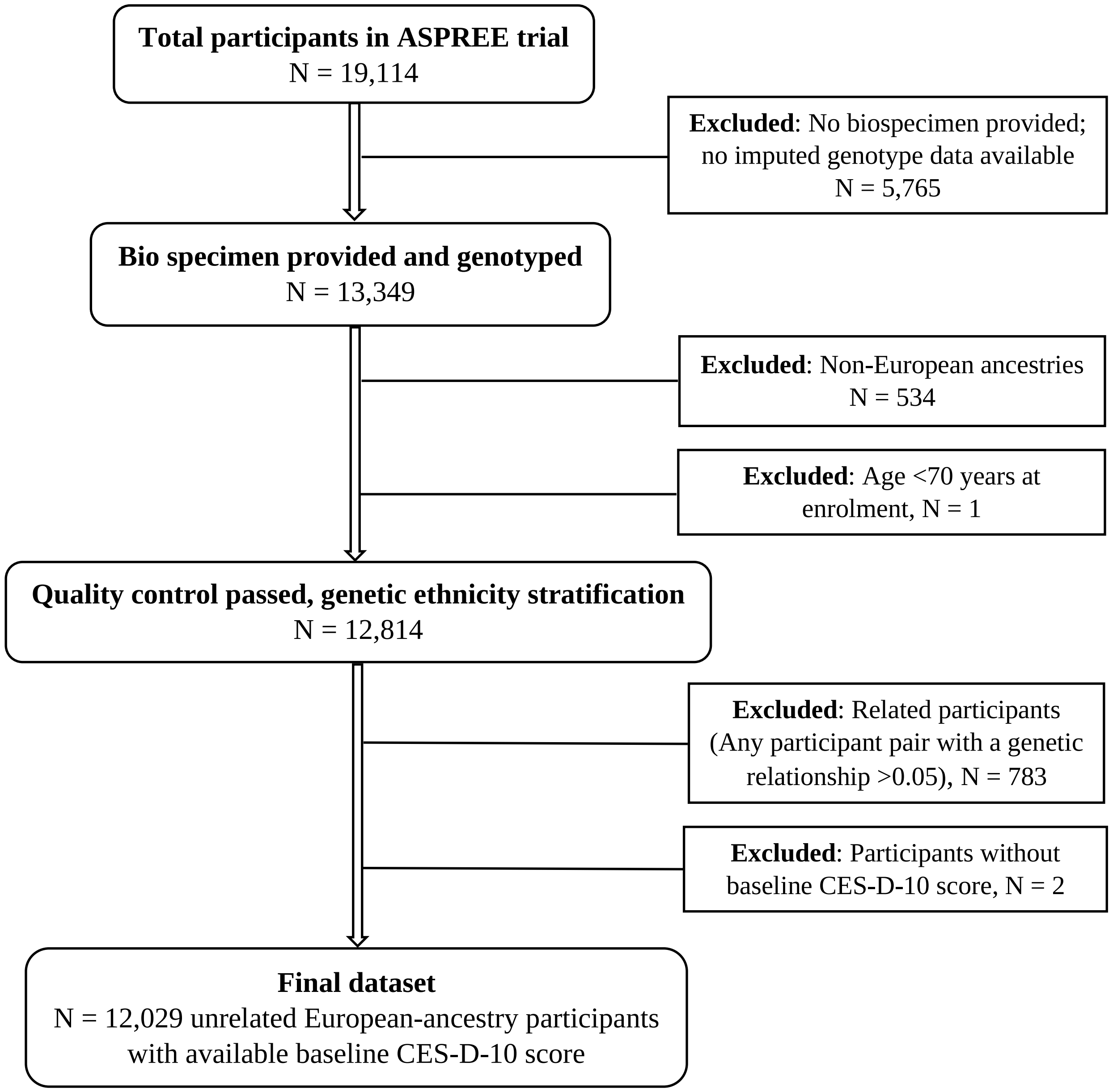

Figure S2: Principal component analyses of genotyped participants in ASPREE and 1000 genomes (1KG) populations (super pops: AFR, AMR, EAS, EUR, and SAS). Three ASPREE populations (Caucasian, Hispanic, and South Asian) are clearly separated and match with the 1000G populations. We focused on the Caucasian participants in this study.

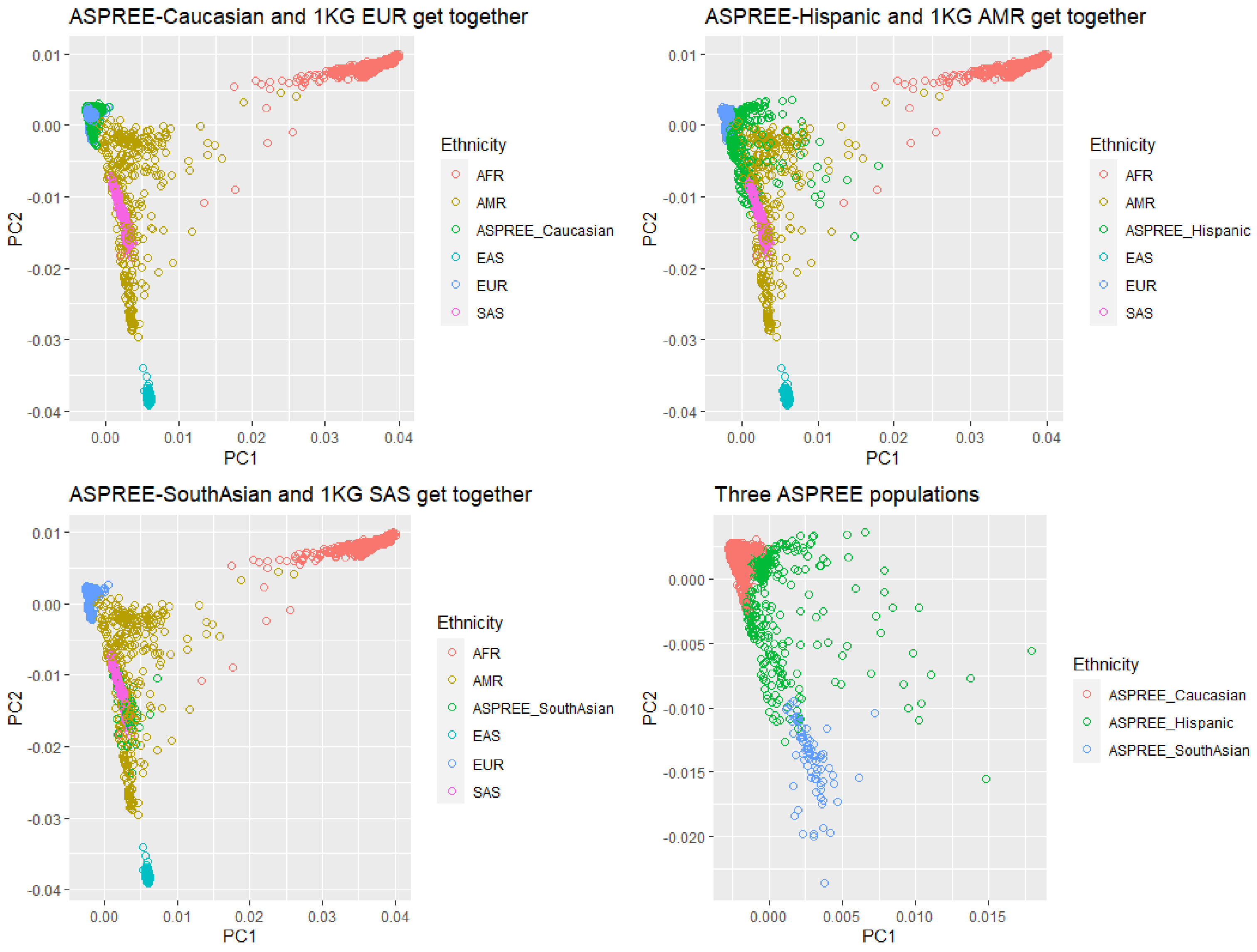

Figure S3: Distribution of baseline CES-D-10 scores (ranging from 0 to 30) of the 12,029 ASPREE participants.

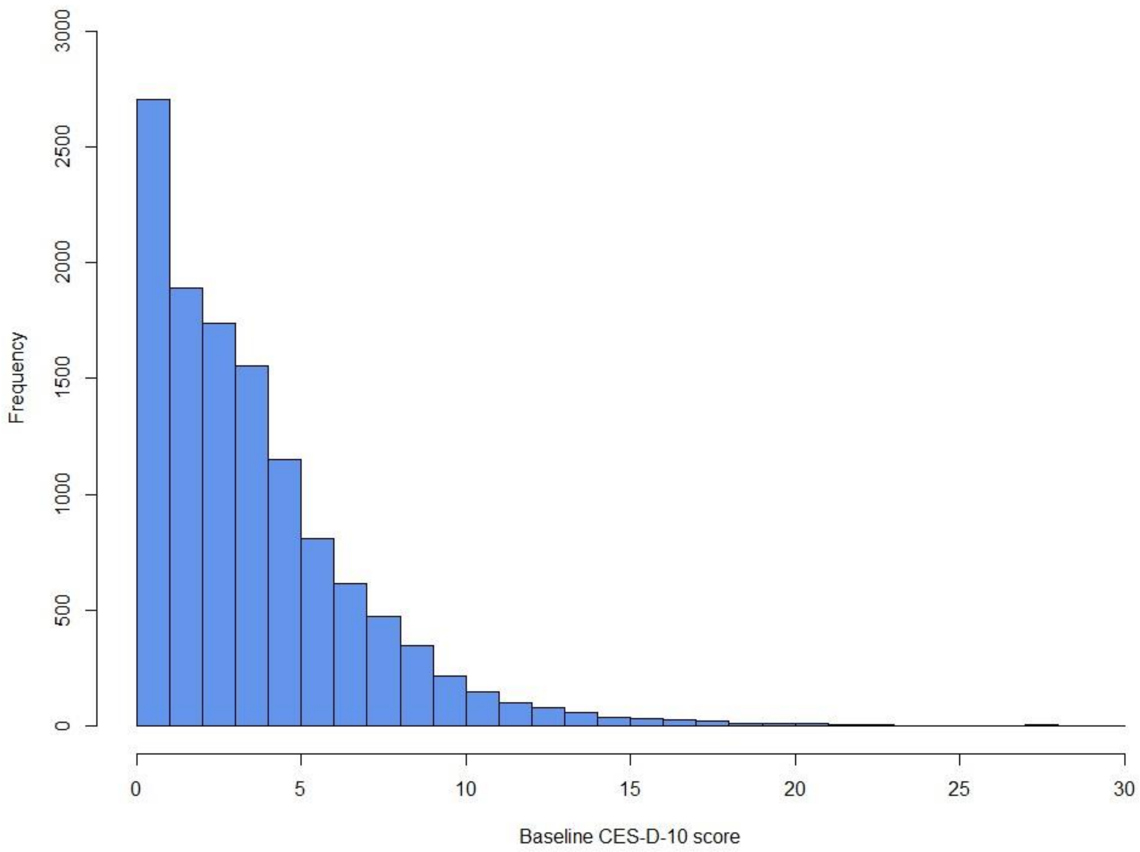

Figure S4. Regression plots comparing variant effect sizes between PGC and ASPREE. Each scatter plot illustrates the relationship between effect sizes (log(odds ratio)) of the top 1,000 significant SNPs in PGC (European descent) and their corresponding effect sizes in ASPREE (adjusted for age, sex and the first 20 PCs) across different CES-D-10 score thresholds (8, 10, and 12). The x-axis represents effect sizes from PGC, while the y-axis represents effect sizes from ASPREE. Each point corresponds to a SNP, with the red line indicating the regression fit. Pearson correlation coefficient *r* and P-values are provided for each threshold, highlighting a significant correlation between effect sizes in the two datasets.

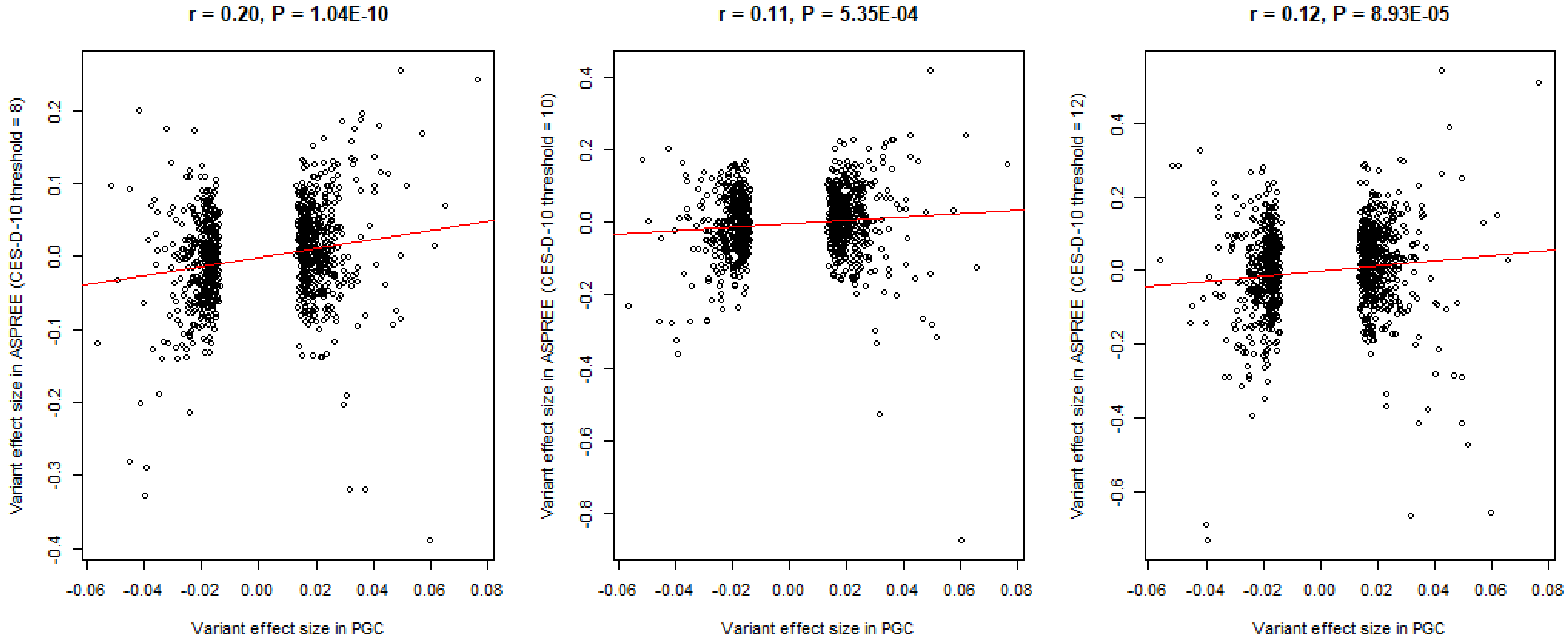

Figure S5: PGS distribution of the 12,029 ASPREE participants (low-risk 0-20%, medium-risk 21-80%, and high-risk 81-100%).

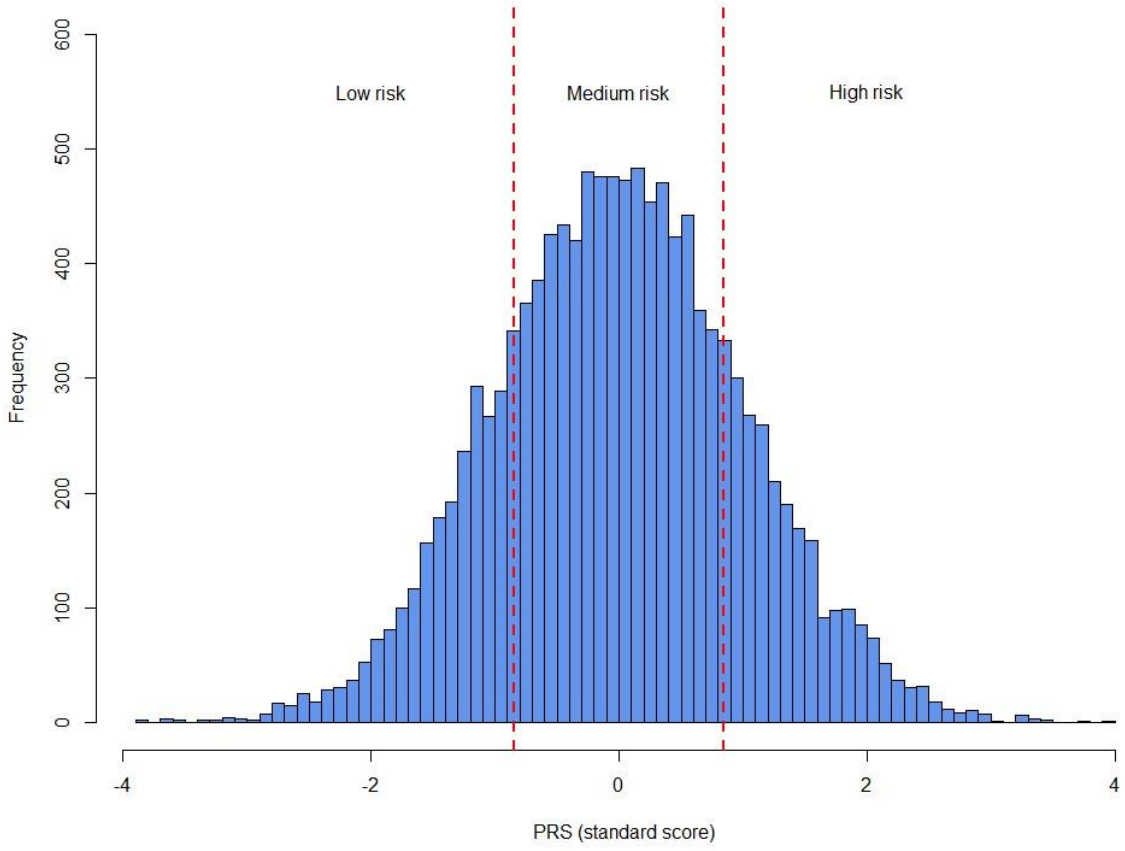

Figure S6: Association between the PGS groups (deciles) and antidepressant medication use at baseline. Logistic Models 1.1 and 1.2 were used to estimate OR and 95% CI. In Model 1.1, we adjusted for age, sex, and the first 20 PCs. Model 1.2 was additionally adjusted for living status, education, smoking status, alcohol drinking status, and BMI.

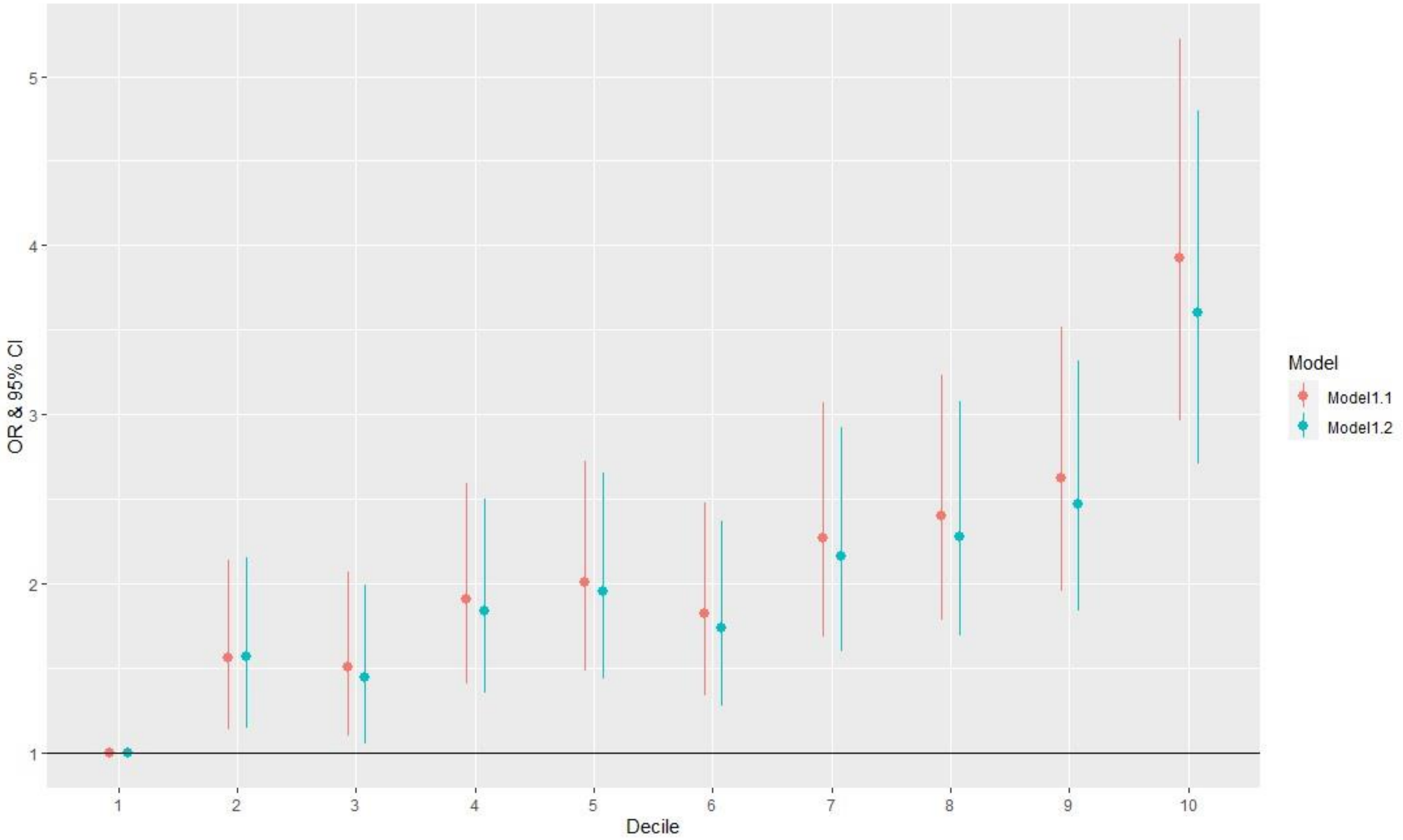

Figure S7: PGS distributions (mean and interquartile range) of four depression trajectory classes. Four distinct classes reflect consistently low (Non-depressed), consistently moderate (Subthreshold depression), consistently high (Persistent depression) and initially low but emerging symptoms of depression (Emerging depression).

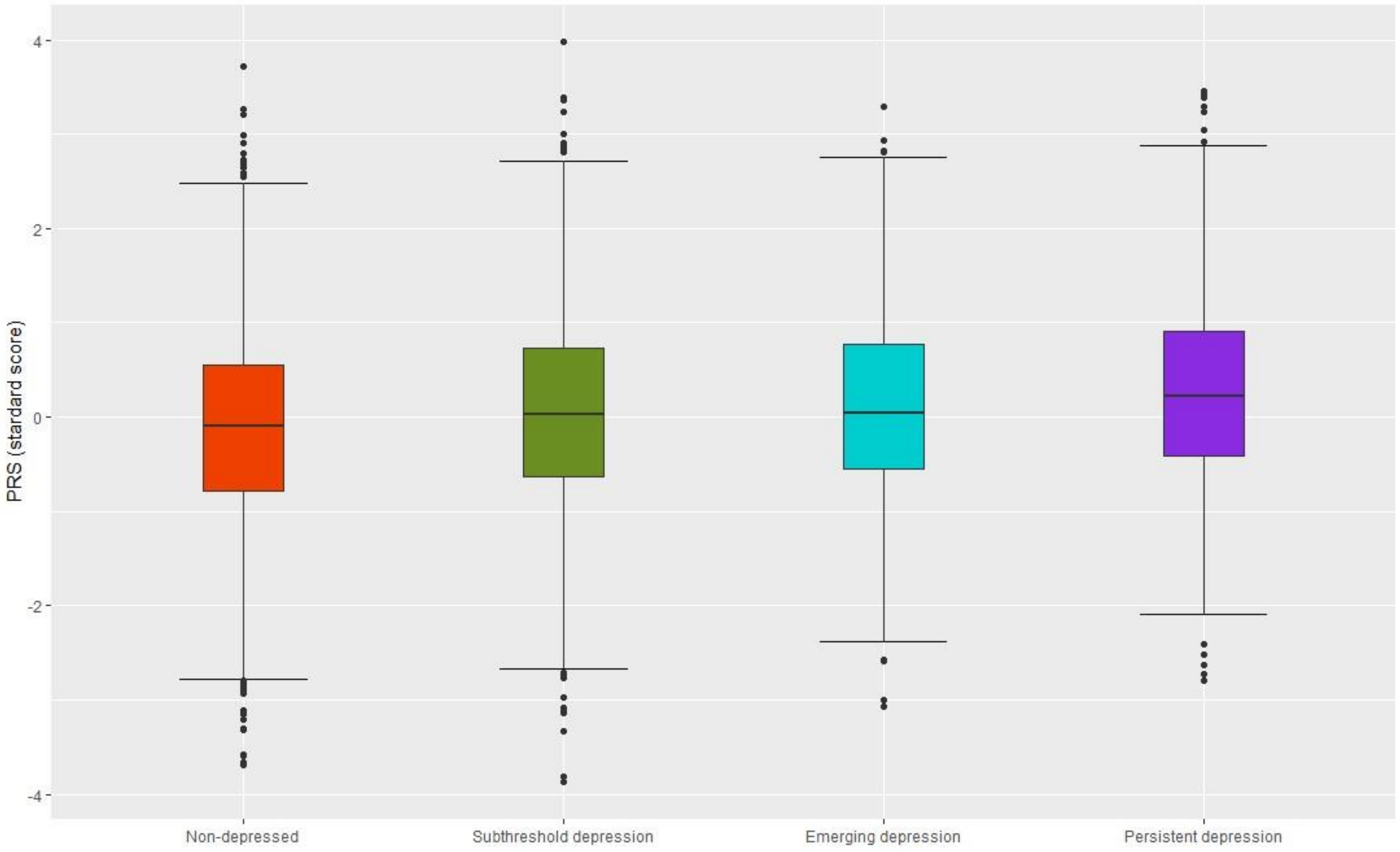

Table S1: Summary statistics of longitudinal CES-D-10 scores of the 12,029 ASPREE participants.

Year	Participant No.	Score Mean (SD)	Participant No. with score ≥8	Participant No. with score ≥10	Participant No. with score ≥12
Baseline	12,029	3.09 (3.20)	1,096	535	291
1	11,707	4.03 (3.91)	1,821	1,057	632
2	4,752	3.87 (3.82)	696	409	230
3	10,084	4.19 (3.99)	1,710	1,015	609
4	6,918	4.12 (3.95)	1,147	685	423
5	4,827	4.35 (3.94)	867	500	295
6	1,802	4.30 (4.02)	322	191	107

Table S2: Association between the PGS (continuous values) and baseline and incident depression with different thresholds of CES-D-10 scores using Models 1.1 and 2.1.

CES-D-10 threshold	Baseline depression				Incident depression		
	Case No.	Control No.	Model 1.1		Incident case No. at follow-ups	Model 2.1	
			OR (95% CI)	P		HR (95% CI)	P
≥8	1,096	10,933	1.26 (1.18 - 1.34)	4.36E-13	3,015	1.20 (1.16 - 1.24)	1.32E-22
≥10	535	11,494	1.32 (1.21 - 1.44)	3.50E-10	2,121	1.25 (1.19 - 1.30)	1.96E-23
≥12	291	11,738	1.41 (1.26 - 1.59)	7.74E-09	1,412	1.33 (1.26 - 1.40)	3.25E-26

Note: Logistic Model 1.1 and Cox Model 1.2 were used to estimate OR/HR and 95% CI per SD. These models were adjusted for age, sex, and the first 20 PCs.

Table S3: AUC estimates and comparisons for risk prediction of baseline depression.

CES-D-10 threshold	AUC (95% CI)		P by DeLong test
	Baseline Model	Baseline Model + PGS	
≥8	0.62 (0.60-0.63)	0.63 (0.61-0.64)	0.02
≥10	0.64 (0.62-0.66)	0.65 (0.63-0.68)	0.02
≥12	0.67 (0.64-0.70)	0.69 (0.65-0.72)	0.09

Table S4: Association between the PGS (continuous values) and antidepressant medication use at baseline.

No. of participants who use	No. of participants who do not use	Model 1.1		Model 1.2	
		OR (95% CI)	P	OR (95% CI)	P
1,345	10,684	1.42 (1.34 - 1.51)	9.15E-33	1.39 (1.31 - 1.47)	1.56E-27

Note: Logistic regression Models 1.1 and 1.2 were used to estimate OR and 95% CI per SD. In Model 1.1, we adjusted for age, sex, and the first 20 PCs. Model 1.2 was additionally adjusted for living status, education, smoking status, alcohol drinking status, and BMI.

Table S5: Association between the PGS (continuous values) and depression trajectory classes using Model 3.1.

Depression trajectory class comparison	Model 3.1	
	OR (95% CI)	P
Non-depressed (reference) vs. Subthreshold depression	1.17 (1.12 - 1.21)	2.33E-14
Emerging depression	1.23 (1.14 - 1.33)	9.20E-08
Persistent depression	1.45 (1.35 - 1.54)	8.35E-28
Subthreshold depression (reference) vs. Emerging depression	1.05 (0.98 - 1.14)	0.18
Persistent depression	1.24 (1.16 - 1.32)	2.81E-10
Emerging depression (reference) vs. Persistent depression	1.18 (1.07 - 1.29)	5.98E-04

Note: Non-depressed, n = 5,536; Subthreshold depression, n = 4,625; Emerging depression, n = 779; Persistent depression, n = 1,089. Multinomial logistic Model 3.1 was used to estimate OR and 95% CI per SD and was adjusted for age, sex, and the first 20 PCs.

Table S6: P-values for PGS (continuous)-by-covariate interaction in depression outcomes using fully adjusted models.

Depression outcome	PGS-by-sex	PGS-by-living status [a]	PGS-by-education	PGS-by-smoking [b]	PGS-by-alcohol [c]	PGS-by-BMI
Baseline depression			Model 1.2			
CES-D-10 threshold ≥8	0.59	0.05	**0.04**	0.21	0.57	0.59
		0.56		0.23	0.59	
CES-D-10 threshold ≥10	0.32	0.84	0.91	0.70	0.31	0.66
		0.87		0.97	0.40	
CES-D-10 threshold ≥12	0.05	0.19	0.41	0.62	0.88	0.77
		0.91		0.83	0.89	
Incident depression			Model 2.2			
CES-D-10 threshold ≥8	0.92	0.96	0.60	**0.04**	0.93	0.94
		0.48		**0.03**	0.36	
CES-D-10 threshold ≥10	0.52	0.47	0.69	0.09	0.22	0.69
		0.99		0.08	0.33	
CES-D-10 threshold ≥12	0.76	0.89	0.22	0.04	0.54	0.65
		0.70		**0.04**	0.34	
Antidepressant medication use at baseline			Model 1.2			
	0.32	0.56	0.16	0.92	**0.02**	0.61
		**0.04**		0.96	0.97	
Depression trajectory class comparison			Model 3.2			
Non-depressed (reference) vs. Subthreshold depression	0.94	0.99	0.98	0.30	0.38	0.77
		0.68		0.39	0.34	
Emerging depression	0.67	0.29	**0.01**	0.21	0.54	0.05
		0.70		0.08	0.79	
Persistent depression	0.34	0.22	0.66	0.48	0.52	0.45
		0.37		0.53	0.08	
Subthreshold depression (reference) vs. Emerging depression	0.64	0.30	**0.01**	0.07	0.27	0.08
		0.90		**0.03**	0.44	
Persistent depression	0.31	0.22	0.65	0.12	0.99	0.34
		0.52		0.18	0.25	
Emerging depression (reference) vs. Persistent depression	0.74	0.97	0.06	0.50	0.33	**0.03**
		0.73		0.24	0.14	

[a] Using "At home alone" as a reference, the two P-values were derived by comparing "At home with family, friends or spouse" and "In a residential home", respectively.
[b] Using "Current smoking" as a reference, the two P-values were derived by comparing "Former smoking" and "Never smoking", respectively.

[c] Using “Current alcohol drinking” as a reference, the two P-values were derived by comparing “Former alcohol drinking” and “Never alcohol drinking”, respectively.
Note: Logistic Model 1.2, Cox Model 2.2 and multinomial logistic Model 3.2 were used to estimate OR/HR and 95% CI per SD, and the models were adjusted for age, sex, the first 20 PCs, living status, educational attainment, smoking status, alcohol drinking status, and BMI.
